# Call for intervention and analysis of the rise in young-onset gastrointestinal cancers in low- and middle-income countries: an editorial

**DOI:** 10.1097/MS9.0000000000001964

**Published:** 2024-03-19

**Authors:** Hareesha Rishab Bharadwaj, Nicholas Aderinto, Syed Hasham Ali, Joecelyn Kirani Tan, Arkadeep Dhali, Khabab Abbasher Hussein Mohamed Ahmed

**Affiliations:** a Faculty of Biology Medicine and Health, The University of Manchester, Manchester; b Academic Unit of Gastroenterology, Sheffield Teaching Hospitals, Sheffield; c School of Medicine and Population Health, The University of Sheffield, Sheffield; d Faculty of Medicine, University of St Andrews, St Andrews, UK; e Internal Medicine Department, LAUTECH Teaching Hospital, Oyo, Nigeria; f Faculty of Medicine, Dow Medical College, Dow University of Health Sciences, Karachi, Pakistan; g Faculty of Medicine, University of Khartoum, Khartoum, Sudan

Highlights

- Growing incidence of early-onset gastrointestinal (EO GI) cancers in low- and middle-income countries (LMICs) is a pressing global health issue.
- Challenges include limited data, underreporting, diagnostic and screening disparities, and infrastructure constraints.
- Positive trends include endoscopic screening programmes and innovative diagnostic strategies, but research funding challenges persist.
- Comprehensive solutions advocated: establish robust cancer registries, integrate technology for real-time reporting, invest in healthcare infrastructure, and foster international collaborations to enhance research capacity.

The surge in early-onset (EO) gastrointestinal (GI) cancers within the age group of 18–49 years demands extensive exploration to comprehend its multifaceted nature^1^. This trend, spanning various GI cancers such as colorectal, extrahepatic bile duct, gallbladder, liver, pancreas, and stomach cancers, suggests a significant shift in the age of diagnosis. The proposed hypothesis implicates a substantially modified early life exposome, encompassing changes in lifestyle, diet, environmental carcinogens, obesity, and prevalent antibiotic exposure in 20th-century living. Further research is imperative to comprehensively elucidate the underlying pathophysiology, particularly concerning the intricate interplay between the microbiome, immune system development, and carcinogenesis^1^.

Despite the classification of this trend as a high-priority research area, the predominant focus has been on high-income countries (HICs)^2^. This emphasises the critical imperative to broaden research initiatives into low- and middle-income countries (LMIC) settings. The current epidemiological data primarily originate from cancer registries in HICs, leaving a considerable gap in our understanding of EO GI cancers in diverse global contexts. This editorial seeks to amalgamate existing research in LMIC settings, advocating for heightened research efforts. It underscores the necessity to recognise and address the unique challenges and characteristics impacting the presentation and management of EO GI cancers in such contexts.

A comprehensive analysis of the research landscape in LMICs does reveal concerted efforts to investigate the prevalence of young-onset GI cancers. Initiatives involve in-depth epidemiological registries aimed at delineating the distinctive characteristics of EO GI cancers. In Brazil, a retrospective analysis unveiled a significant prevalence of EO GI tract malignancies, with a relative increase of 35% in incidence^3^. In China, a concerning total of 681 178 new cases were reported in 2017 alone, and liver cancer notably emerged as the primary contributor to cancer-related mortality among young adults during that year^4^. Likewise, India demonstrated a significant increase in EO cases, characterised by heightened clinical aggressiveness^5^. For instance, young individuals diagnosed with rectal cancer exhibited a higher incidence of poorly differentiated cancer at 36.8%, in contrast to 15.78% observed in the older age group^5^. Notably, the economic conditions prevailing in the country of residence appear to influence the occurrence of EO colorectal cancers (CRCs)^6^. Young Nigerian patients demonstrated a higher frequency of EO CRCs in comparison to their counterparts in the African American population^6^.

Efforts at diagnosing EO GI malignancies in LMICs include the implementation of endoscopic screening programmes similar to those in higher-income counterparts. For instance, China’s Upper Gastrointestinal Cancer Early Detection (UGCED) initiative focuses on endoscopic screening for EO oesophagal and gastric cancers^7^. Researchers in certain LMICs, such as China, have investigated cost-effective strategies for risk stratification in EO CRC, developing a faecal immunochemical test (FIT)-based risk-stratification model, thereby shedding light on efforts taken by LMIC nations to increase diagnosis of EO GI cancers^8^. Efforts to enhance early diagnosis of EO liver carcinoma through alpha-fetoprotein (AFP) have also been performed in LMICs like Mongolia^9^. In parallel with epidemiological registries and efforts to enhance EO GI cancer diagnosis, specific LMICs have actively explored the intricate connections between environmental factors and potential risk factors associated with EO GI cancers. Researchers in Egypt, for instance, have scrutinised the link between elevated rates of EO pancreatic cancers in the East Nile Delta region. Additionally, active EO cancer prevention strategies have been developed, targeting specific epidemiological causes, such as removing structural barriers to accessing GI care for liver and CRCs^10^.

Despite the above-mentioned positive trends, tackling EO GI cancers in LMICs requires a plethora of hurdles to be addressed. Limited data and underreporting present substantial challenges in understanding the landscape of EO GI cancers within LMICs. The dearth of comprehensive and reliable data is attributed to underreporting, inadequate healthcare infrastructure, and deficient surveillance systems^11,12^. This data deficiency is exacerbated by the absence of well-established cancer registries in many LMICs with only about 21% of the global population covered by such registries. This disparity is particularly pronounced in Asia (8%) and Africa (11%), hindering the formulation of effective strategies and interventions for EO GI cancers. Furthermore, low awareness and limited access to cancer care in LMICs contribute to late presentations, misdiagnoses, and underdiagnoses^13^.

Diagnostic and screening disparities further compound the challenges. Limited access and affordability of crucial diagnostic tools, coupled with the scarcity of well-established screening programmes, contribute to late-stage diagnoses in LMICs. These delays not only impact the effectiveness of treatments but also impede a comprehensive understanding of the prevalence and specific characteristics of EO GI cancers in these regions^14^. Infrastructure and resource constraints in LMICs constitute another formidable obstacle. The inadequate healthcare infrastructure, shortage of specialised cancer treatment centres, and a deficit in trained medical professionals, particularly GI cancer specialists, contribute to suboptimal care. Only 8% of oncology randomised clinical trials in LMICs, as highlighted by Rubagumya *et al*.^15^, underscore the lack of research capacity and expertise in addressing GI cancers in these regions. Research funding challenges further limit the pursuit of knowledge and effective solutions. Gopal (2023) emphasises the financial constraints hindering the development of innovative strategies and interventions^16^. This limitation harbours the potential to impede the identification of critical aspects such as disease aetiology, prevalence patterns, and treatment outcomes, thus hampering progress in prevention, early detection, and patient care.

Addressing these challenges in LMICs requires a comprehensive approach and a strategic plan for future research and intervention efforts. Consequently, the authors advocate that subsequent initiatives should focus on establishing robust cancer registries in LMICs, collaborating with international organisations, and sharing best practices. Integration of technology, such as digital health platforms, could facilitate real-time reporting, enhancing the accuracy and completeness of data on EO GI cancers. Investment in infrastructure and medical technology is crucial to overcome diagnostic disparities. Future efforts should prioritise establishing and upgrading healthcare facilities with advanced diagnostic capabilities. Public health campaigns aimed at raising awareness about the importance of early diagnosis and available screening programmes can contribute to reducing diagnostic delays.

Long-term strategies should include substantial investments in building and strengthening healthcare infrastructure in LMICs. This involves expanding the number of specialised cancer treatment centres and training a skilled oncology workforce. Collaboration with international partners and organisations can facilitate knowledge transfer, training programmes, and the establishment of comprehensive cancer care facilities. Future prospects should prioritise building research capacity in LMICs and fostering local expertise in the field of EO GI cancers. Initiatives such as research training programmes and collaborative projects can enhance the ability of LMICs to lead research efforts. Encouraging the participation of LMIC researchers in international clinical trials and research collaborations can further contribute to expanding the knowledge base.

In conclusion, the burgeoning incidence of EO GI cancers in LMICs necessitates a collective and sustained effort to address challenges and explore future prospects. The complex interplay of limited data, diagnostic disparities, socio-economic barriers, and treatment inequalities underscores the urgency of a multifaceted approach. By investing in research, healthcare infrastructure, and international collaboration, we can pave the way for a comprehensive understanding and effective management of EO GI cancers in LMICs.

## Ethical approval

Ethics approval was not required for this Editorial Article.

## Consent

Informed consent was not required for this Editorial Article.

## Sources of funding

This study received no funding or financial support.

## Author contribution

Conceptualization, data curation, writing the original draft, review, and editing: H.R.B., N.D., S.H.A., J.K.T., and A.D.; supervision, reviewing, and editing: K.A.H. and H.R.B.

## Conflicts of interest disclosure

The authors declare they have no conflicts of interest to declare.

## Research registration unique identifying number (UIN)

1. Name of the registry: not required.
2. Unique identifying number or registration ID: not applicable.
3. Hyperlink to your specific registration (must be publicly accessible and will be checked): not applicable.

## Guarantor

Khabab Abbasher Hussien Mohamed Ahmed, Faculty of Medicine, University of Khartoum, Khartoum 11111, Sudan. E-mail: khabab9722@gmail.com, ORCID: https://orcid.org/0000-0003-4608-5321.

## Data availability statement

The data that support the findings of this study are available with the corresponding author upon reasonable request.

## Provenance and peer review

Not commissioned, externally peer-reviewed.
